# Intravital microscopy datasets examining key nephron segments of transplanted decellularized kidneys

**DOI:** 10.1038/s41597-022-01685-9

**Published:** 2022-09-10

**Authors:** Peter R. Corridon

**Affiliations:** 1 Department of Immunology and Physiology, College of Medicine and Health Sciences, Khalifa University of Science and Technology, PO Box 127788, Abu Dhabi, UAE; 2 Wake Forest Institute for Regenerative Medicine, Medical Center Boulevard, Winston-Salem, NC 27157-1083 USA; 3 Biomedical Engineering, Healthcare Engineering Innovation Center, Khalifa University of Science and Technology, PO Box 127788, Abu Dhabi, UAE; 4 Center for Biotechnology, Khalifa University of Science and Technology, PO Box 127788, Abu Dhabi, UAE

**Keywords:** End-stage renal disease, Biological fluorescence, Multiphoton microscopy

## Abstract

This study contains intravital microscopy (IVM) data examining the microarchitecture of acellular kidney scaffolds. Acellular scaffolds are cell-free collagen-based matrices derived from native organs that can be used as templates for regenerative medicine applications. This data set contains *in vivo* assays that evaluate the effectiveness of decellularization and how these acellular nephron compartments perform in the post-transplantation environment. Qualitative and quantitative assessments of scaffold DNA concentrations, tissue fluorescence signals, and structural and functional integrities of decellularized tubular and peritubular capillary segments were acquired and compared to the native (non-transplanted) organ. Cohorts of 2–3-month-old male Sprague Dawley rats were used: non-transplanted (n = 4), transplanted day 0 (n = 4), transplanted day 1 (n = 4), transplanted day 2 (n = 4), and transplanted day 7 (n = 4). Micrographs and supporting measurements are provided to illustrate IVM processes used to perform this study and are publicly available in a data repository to assist scientific reproducibility and extend the use of this powerful imaging application to analyze other scaffold systems.

**Table:** 

Measurements(s)	DNA quantification • tissue fluorescence • microvascular leakage • tubular and peritubular capillary integrity
Technology Type(s)	intravital microscopy • multiphoton microscopy • UV-visible spectroscopy
Sample Characterization(s)	rats • native and decellularized kidneys

## Abstract (summary)

**Table:** 

Measurement(s)	DNA • tissue fluorescence
Technology Type(s)	ultraviolet-visible spectrophotometry • intravital microscopy
Sample Characteristic - Organism	mouse/rat NG108-15
Sample Characteristic - Environment	laboratory environment
Sample Characteristic - Location	contiguous United States of America • Emirate (United Arab Emirates)

## Background and Summary

The global rise in organ failure and scarcity of transplantable organs has heightened the pursuit of clinical alternatives. Within the past decade, advances in whole organ bioengineering technologies have demonstrated the potential to create such options. Decellularization is at the forefront of these technologies, as this method separates the extracellular matrix (ECM) from the native cells^1^. The ECM, with its preserved embedded structural and functional cues, can be employed as a natural and ideal scaffold to produce functioning replacement organs^2^. Such templates have been created from human- and animal-derived blood vessels^3^ and tracheas^4^, and more complicated structures, including the heart, lung, liver, and kidney^5^, as well as eyes^6^.

Presently, the outcomes used to identify the efficacy of the decellularization process rely on the absence of cellular or nuclear remnants from the well-preserved tissue structure and maintenance of mechanical properties comparable to native tissues. The protocol’s effectiveness also depends upon the size of the original organ/tissues, the concentration of the decellularization agent (typically detergents), and the perfusion rate. For instance, several reports have shown how anionic detergents like sodium dodecyl sulfate (SDS) can routinely remove cells and genetic material from native structures quickly and effectively to produce homogeneous whole acellular renal templates^1,7–12^. These acellular scaffolds, primarily made from solid organs, are often created from perfusion with this single surfactant or a blend of ionic and non-ionic surfactants. Nevertheless, a significant challenge to this process lies within the decellularized vasculature of complex organs like the kidney. The ideal conditions that will support scaffold longevity within transplantation environments are still unknown, as there is a limited understanding of decellularized microarchitectural dynamics *in vivo*^13^.

To this end, across the years a variety of microscopic and macroscopic approaches have been used to evaluate primary scaffold constituents^14–16^, as well as whole kidney scaffold structure and function^9,10,13,17–20^. In several animal models, such techniques have shown that the decellularization process successfully removes native cellular components while retaining the vascular and ECM structures. The vascular architecture must be preserved at the microscopic level because it serves as an effective conduit to facilitate recellularization (the reintroduction of cells into scaffolds) and maintain organ viability post-transplantation. Furthermore, the efficient removal of scaffold remnants is critical for preventing undesirable biological and immunological consequences during recellularization and following transplantation. Current approaches, on the other hand, including confocal imaging, histology, and electron microscopy^20^, as well as micro-CT imaging^9^, are unable to support *in vivo* investigations with the required spatial and temporal resolutions. These limitations highlight the need for new ways to examine microarchitectural dynamics within scaffolds post-transplantation.

Interestingly, intravital microscopy (IVM) provides a unique opportunity to assess live morphological and functional aspects, particularly within the renal tubules and peritubular cappilaries^17–20^. Consequently, the objective of this investigation was to obtain real-time *in vivo* data on the decellularized microarchitecture of these key nephron segments after transplantation using IVM. Techniques from previously refined methods that protect the existing vascular network while limiting scaffold toxicity were applied to generate acellular scaffolds^9,20^. In this case, intact rat kidneys were extracted and decellularized by perfusion with SDS. The resulting scaffolds were orthotopically implanted into their respective donors. The removal rate of cellular remnants and the viability of the decellularized network were then confirmed in real-time using nuclear and vascular dyes, respectively.

**Fig. 1:**
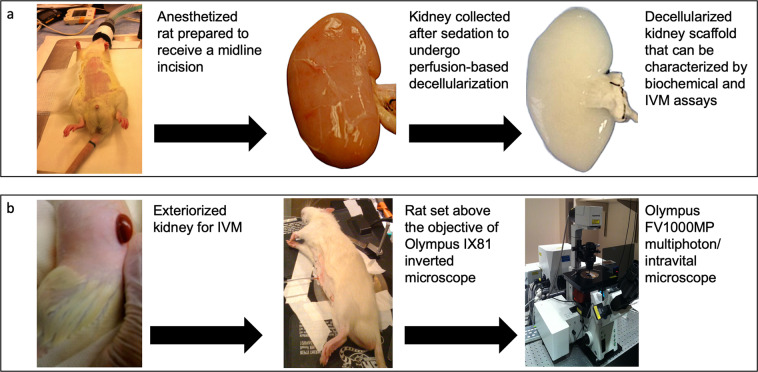
Overview of the study design. Photographs taken to illustrate (**a**) animal and organ manipulations to create acellular scaffolds for characterizations and (**b**) animal and organ preparations for IVM data acquisition and analysis.

This data set also contains results from *in vivo* assays that assessed the effectiveness of the decellularization process and the structural and functional integrities of vital acellular nephron segments in the post-transplantation environment. Qualitative and quantitative assessments of tissue structure (via autofluorescence) and tubular and capillary integrities (via blood extravasation) were acquired in live rats on day 0, day 1, day 2, and day 7 after transplantation and compared to data obtained from the native (non-transplanted) organs. This sophisticated imaging technology’s ability to evaluate renal dynamics *in vivo* is extensively documented^17,18,21–36^. The manuscript highlights methods of monitoring cellular/subcellular processes in real-time within transplanted kidney scaffolds, which can be extended to other organ scaffold systems. An overview of the experimental approach can be found in Fig. 1.

## Methods

### Study design

This manuscript presents data from a time-course study used to examine acellular kidney scaffolds using IVM. Experiments were conducted on 2–3-month-old male Sprague Dawley rats. Each animal was randomly assigned to one of the following cohorts: non-transplanted (n = 4; n = 1 day 0, n = 1 day 1, n = 1 day 2, and n = 1 day 7); transplanted day 0 (n = 4); transplanted day 1 (n = 4); transplanted day 2 (n = 4); and transplanted day 7 (n = 4). An additional group of 4 rats was used for technical validation of the decellularization process, whereby both kidneys from each animal were used for biochemical assays to evaluate DNA and SDS concentrations in native (n = 4) and decellularized (n = 4) kidneys. The animals from that group received bilateral nephrectomies and were sacrificed after this procedure. The remaining animals that received unilateral nephrectomies were tracked by drawing color-coded lines around their tails with permanent markers and recording their associated cage numbers. Animal identities were also duplicated on cage cards and laboratory records. The experimenter was not blinded to the animal’s identity throughout the entire experimental and analysis periods, and no animal was excluded from this study.

At the start of the study, urine creatinine levels were monitored, and nephrectomies were performed to obtain whole kidneys to create the decellularized scaffolds. Transplantations and IVM studies were conducted after the kidneys were decellularized and the animals had enough time to recover from the radical left nephrectomy. Biochemical assays were performed to evaluate DNA and SDS contents during the recovery period. All experiments were performed under the Institutional Animal Care and Use Committee at Wake Forest University’s School of Medicine (Winston-Salem, NC, USA), the Animal Research Oversight Committee at Khalifa University of Science and Technology (Abu Dhabi, UAE), and ARRIVE criteria. These experimental procedures are outlined in detail below.

### Urine creatinine measurements

**Fig. 2:**
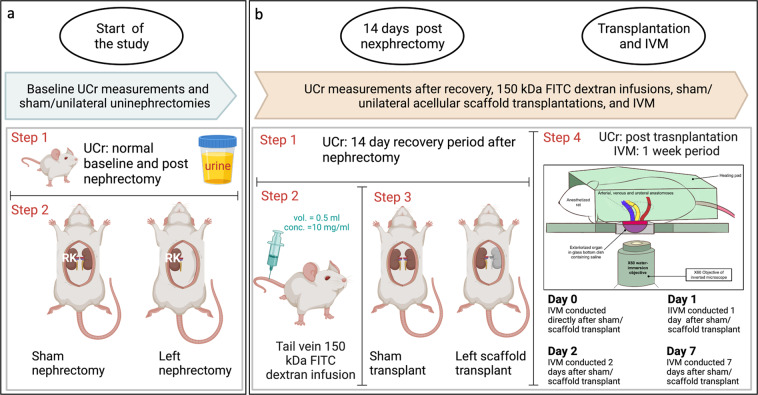
Schematic of the data acquisition timeline. (**a**) An outline of the events that occurred at the start of the study including baseline urine creatinine (UCr) monitoring, sham/unilateral nephrectomies. (**b**) Series of urine and IVM measurements that occurred across the 7-day period.

Animals were placed in individual metabolic cages to enable 24-hour urine collections. From these samples, urine creatinine (UCr) was measured using a Beckman Synchron CX 5 CE analyzer (Beckman Coulter Inc., Brea, CA). UCr levels were monitored at the start of the study directly after unilateral/sham nephrectomies, 14 days after recovery from unilateral/sham nephrectomies, and across the 7 days after sham/unilateral acellular scaffold transplantation (Fig. 2). The dataset (Urine_creatinine_assay.csv) shows that UCr levels were in most cases slightly reduced after nephrectomies. However, these values dropped sharply in these animals after they were transplanted with acellular scaffolds indicating impaired renal function following transplantation of the decellularized kidneys.

### Nephrectomies

Sham, unilateral, and bilateral nephrectomies were performed on 2–3-month-old male Sprague Dawley rats (Envigo, Indianapolis, IN, USA). These animals were initially exposed to isoflurane (Webster Veterinary Supply, Devens, MA, USA) and then injected with 50 mg/kg pentobarbital intraperitoneally and placed on a heating pad to regulate core body temperature, as shown in Fig. 1. After that, midline incisions were then created along the aseptic regions, defined by the topical application of Betadine Surgical Scrub (Purdue Products L.P., Stamford, CT, USA) to expose the left kidney. The corresponding renal artery, renal vein, and ureter were ligated with 4-0 silk (Fine Science Tools, Foster City, CA, USA). The native kidneys were extracted with intact segments of the renal arteries, veins, and ureters, as shown in Fig. 1. The incision was closed in animals that received unilateral nephrectomies, and all animals were given two weeks to recover. Similar midline incisions and organ collection processes were performed during bilateral and sham nephrectomies.

### Scaffold decellularization, sterilization, and storage

The artery of each extracted kidney was connected to a 14-gauge cannula (Fine Science Tools, Foster City, CA, USA) and PE-50 polyethylene catheter tubing (Clay Adams, Division of Becton Dickson, Parsippany, NJ, USA) through which heparinized PBS (0.5–1 mL) was immediately perfused. The organs were then immersed in PBS, and the cannulated renal artery was connected to a peristaltic pump (Cole-Palmer, Vernon Hills, IL, USA). The kidneys were then perfused with 0.5% SDS (Sigma-Aldrich, St. Louis, MO, USA) at a rate of 4 ml/min via the renal artery for a total of six hours, followed by phosphate-buffered saline (PBS) perfusion for 24 hours and 10.0 Ky gamma irradiation. The sterilized scaffolds were then kept at 4 °C.

### Preparation of *in vivo* nuclear staining dyes

The nucleic acid stain, Hoechst 33342 (Invitrogen, Carlsbad, CA, USA), was used to reveal cellular nuclei. Similarly, this infusate was prepared by diluting approximately 50 μl of this cell-permeant probe in 0.5 ml sterilized saline.

### Preparation of large molecular weight fluorescent dextrans infusates

150-kDa fluorescein isothiocyanate (FITC)-dextrans (TdB Consultancy, Uppsala, Sweden), dextrans were used to visualize the microvasculature of the native and decellularized kidneys. A stock solution of this dye was first prepared by mixing 20 mg in 1 ml of sterilized saline. Then the infusate was prepared by diluting 500 μl of the stock solution in 1 ml of sterilized saline.

### Intravital imaging of native and decellularized tubular and peritubular compartments using a multiphoton microscope

After anesthetizing each animal, its thoracic region was again shaved, and the topical antiseptic was applied to this area. The animal’s tail vein was then dilated by either placing it in a warm bath or soaking it with a warm, damp gauze sheet. After identifying the dilated vein, a 25-gauge butterfly needle (Sigma-Aldrich, St. Louis, MO, USA) was inserted into this vascular track to facilitate the infusion of a bolus of 0.5 mL of heparinized saline.

Each rat was then transplanted with a scaffold created from its nephrectomized kidney. For this process, flank incisions were then made, and orthotropic transplantations of the biocompatible acellular scaffolds were achieved by performing end-to-end anastomoses of decellularized renal artery and vein to the rat’s previously ligated renal artery and vein, respectively. Anastomoses were performed by occluding the ligated ends of the vessels with micro-serrefine vascular clamps (Fine Science Tools, Foster City, CA, USA) and connecting the respective opened ends of the scaffold decellularized vessels with 10-0 silk sutures (Fine Science Tools, Foster City, CA, USA). The ureter was also anastomosed to a portion of the renal vein. Fluorescent markers were then infused through the tail vein, and the venous vascular clamp was first removed, followed by the arterial vascular clamp to reperfuse the scaffold. The sites of anastomoses were carefully inspected and additional sutures were added to eliminate any bleeding that was observed.

The native and transplanted organs were exteriorized for imaging at different measurement time points, as shown in Fig. 1b. Exteriorized native or acellular kidneys were individually positioned inside a 50 mm glass-bottom dish (Willco Wells B.V., Amsterdam, The Netherlands) containing saline and a heating pad was put over the animal to maintain body temperature^27^. Images were then obtained with an Olympus FV1000MP multiphoton/intravital microscope (Center Valley, PA, USA) with a Spectra-Physics MaiTai Deep See laser (Santa Clara, CA, USA), Fig. 1b. The laser was tuned to wavelengths ranging from 770 to 860 nm, which are known to excite the fluorescent dyes used in this study. Images were captured using an X60 water-immersion objective and external blue- and green-based emission detectors.

### Biochemical assay to measure decellularization efficacies

Using a Fast Prep 24 Tissue Homogenizer (MP Biochemicals, Santa Ana, CA, USA), the acellular whole organs were homogenized, and 1 ml of methylene blue solution (methylene blue 0.25 g/l, anhydrous sodium sulfate 50 g/l, and concentrated sulfuric acid 10 ml/l) was added to the suspension. The suspension was then digested at 56 °C with proteinase K (Omega Bio-tek, Atlanta, GA, USA) for approximately 1 hour. The samples were then isolated with chloroform, and the SDS content of the extracts was determined using a Molecular Devices SpectraMax M Series Multi-Mode Microplate Reader (Sunnyvale, CA, USA) to obtain absorbance measurements at a wavelength of 650 nm. Concurrently, DNA contents were also quantified using a Qiagen DNeasy Kit (Qiagen, Valencia, CA, USA) by first storing samples of the minced sections at −80 °C overnight. Thereafter, the tissues were lyophilized, and the ratios of ng DNA per mg dry tissue were estimated using an Invitrogen Quant-iT PicoGreen dsDNA assay kit (Carlsbad, CA, USA) and a microplate reader.

**Fig. 3:**
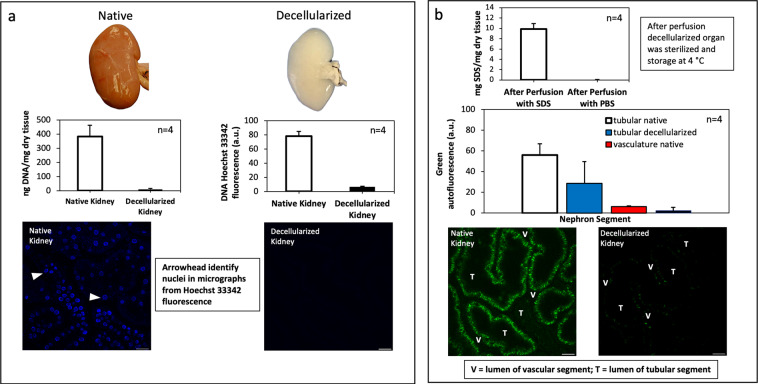
Biochemical and IVM assays that evaluated the decellularization efficacy. (**a**) Graphical displays of DNA concentrations (ng DNA/mg dry tissue) in native and acellular kidneys from biochemical assay correlated with Hoechst 33342 signals (a.u.) in IVM micrographs from fluorescently labeled DNA (arrowheads) in these tissue types. (**b**) Graphical illustrations of SDS removal from scaffolds with PBS and changes in tissue fluorescence with decellularization using IVM micrographs. Kruskal-Wallis tests detected significant DNA, SDS, and tissue fluorescence after decellularization (*p < 0.001).

Biochemical estimations of DNA and SDS contents in native and decellularized kidneys, presented in Fig. 3, are provided to highlight the removal of approximately 90–99% of the innate DNA and SDS contents from the original kidneys post decellularization, as outlined in the literature^13,37^. These results are provided to illustrate the desired efficacy of the decellularization protocol. It should be noted that it is vital to eliminate cellular remains from scaffolds efficiently to limit the risk of undesired immunological responses or tissue rejection following transplantation^13^. A critical feature of the decellularization process is determining the concentrations of these remaining components. It is also critical that residual SDS concentrations are kept low enough to prevent the detergent from further denaturing the scaffold. This undesired action can deleteriously change the scaffold’s overall permeability and impair its integrity, making recellularization and transplantation more difficult. The efficient removal of SDS will also eliminate its potential transfer from the scaffold to the host.

### Intravital microscopy assay to measure decellularization efficacies

**Fig. 4:**
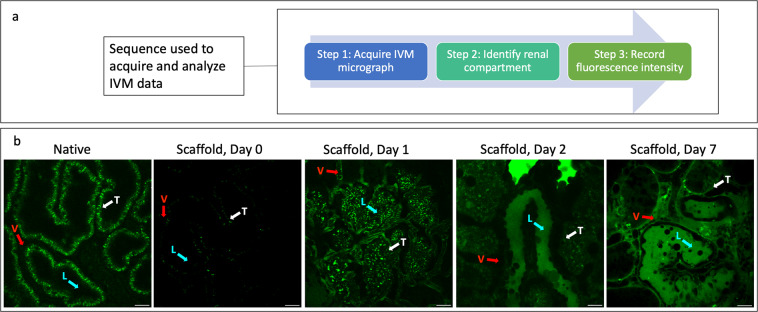
*In vivo* Imaging acquisition. (**a**) A sequence of the IVM data allocation and analysis processes. (**b**) Representative IVM micrographs of native kidneys and scaffolds illustrating regions (arrows in each micrograph) to record fluorescence intensities of the vasculature (V), tubular epithelium (T), and tubular lumen (L). Decellularization would have supported unregulated blood flow throughout the nephron and direct interactions between blood and collagen, resulting in thrombosis, ischemia, and enhanced microarchitectural permeability.

Providing the scientific community with datasets interpreted by experts will support the development of strategies that can advance whole organ bioengineering. To this end, key aspects of the imaging and analysis processes are provided for this study. From multi-photon microscopic data collected, IVM estimations of DNA contents in native and decellularized kidneys provide qualitative and quantitative assessments of *in vivo* tissue fluorescence patterns (Fig. 3a). Likewise, images collected from the green pseudo-color channel revealed differences in the intrinsic autofluorescence patterns in native kidneys compared to the decellularized organs (Fig. 3b). Representative micrographs from day 0 (after transplant), day 1 (after transplant), day 2 (after transplant), and day 7 (after transplant) from cortical regions of native kidneys and scaffolds (outlined in Fig. 4) provide a basis to investigate tissue fluorescence and microvascular integrity/function using well-established image analysis techniques^18,22,25–31,33,38–41^. To conduct these analyses in this instance, randomly selected within a microscopic field, the blue or green pseudo-color fluorescence levels in nuclear and epithelial compartments were measured, respectively.

### Assessments of structural/functional integrities of the decellularized microarchitecture

FITC-dextran infusates with a volume of 0.5 ml and a concentration of 10 mg/ml were introduced into each animal before transplantation. Again, images were taken from cortical regions of native kidneys and scaffolds at the various timepoints, outlined in Figs. 2 and 4, during the week following transplantation to examine peritubular capillary permeability and tubular integrity. One way of examining these characteristics is to estimate the average extravasation rates were calculated across the 7-day experimental period. The average extravasation rate was calculated by taking the mean fluorescence value, at a given timepoint, for each renal compartment and dividing that by the number of hours elapsed after transplantation, which are presented in normalized manner in this data set. The average extravasation rates within these decellularized nephron segments were observed after the 150-kDa FITC dextran was infused into the tail vein before blood was introduced into the transplanted scaffolds. This infusion regimen was chosen over trying to perform infusions after transplantation since anticipated thrombosis could prevent the dye from reaching the microvasculature at later time points. This method allowed the assessment of the distribution of blood contents and insights into the grafts’ ability to withstand *in vivo* conditions.

### Statistical analysis

Statistical analyses were conducted using IBM SPSS Statistics, Mac OS version (IBM Corp, Armonk, NY, USA), whereby a p < 0.05 was considered significant.

## Data Records

The data is located on a publicly available data Figshare repository called “Intravital microscopy datasets examining key nephron segments of transplanted decellularized kidneys,” with the following Digital Object Identifier 10.6084/m9.figshare.19409903^42^. The dataset includes raw data from the DNA biochemical-based (DNA_Biochem_assay.csv) and IVM-based (2P_microscopy_DNA_assay.csv) assays, residual SDS concentration assay (SDS_biochem_assay.csv), and urine creatinine measurements (Urine_creatinine_assay.csv). Files containing the specific methodological details and sample analyses on IVM techniques are also included to illustrate ways to assess the structural and functional integrities of vital acellular nephron segments in the post-transplantation environment, related to both qualitative and quantitative assessments of tissue structure (via autofluorescence) and tubular and capillary integrities: dataset usage notes (IVM_usage_notes.csv), a listing of sample (.TIFF) and (.OIF) micrographs (Micrographs.csv), IVM fluorescence intensities used to view variations between native and decellularized scaffolds (Changes_in_fluoresecnce.csv), sample summarized variations across the 7 day measurement period post FITC infusion (Average_fluoresecence_Day_1_to_7.csv) more detailed measures at each measurement point (Day1_data.csv, Day2_data.csv, and Day7_data.csv), and average extravasation rates (Normalized_extravasation.csv). A PowerPoint (A Guide to Perform IVM Analyses.pptx) is also provided to present a simple step-by-step guide on techniques used to calculate fluorescence intensities from the raw data that can be used to compute degrees of dye extravasation from the vascular lumen.

## Technical Validation

Data provided have been utilized to highlight characterizations of the decellularized whole kidney scaffold prior to recellularization with IVM in a previous study by the author. Several measures were taken to ensure reliability and that the collected data was unbiased. Rats were assigned at random and housed in the same cage. They were all the same breed and provided the same feeding conditions. Additionally, rats were checked for illness or infection throughout the study. All surgical procedures and decellularization techniques were conducted in the same manner, and IVM studies were conducted using the same microscope settings.

For IVM studies, it is well established that the kidney typically has a high level of green autofluorescence^18,39^, caused by biological structures like mitochondria and lysosomes^30^, as well as unique metabolites such as aromatic flavins, nicotinamide adenine dinucleotide, and amino acids^28,43^, emitting light naturally. Without fluorescent markers, the relative distribution and presence of the above structures help routinely identify renal tubular (primarily proximal and distal tubules) and peritubular compartments using this imaging technique^18,22,23,27–30,39^. As a negative control, the infusates of purely saline solution were used to validate the level of fluorescence responses in nuclear, cytoplasmic, vascular, and luminal components. In comparison, the membrane-permeant dye, Hoechst 33342, quickly penetrates tissues where it identifies DNA in living and fixed cells by binding to adenine-thymine-rich sections of DNA in the minor groove, resulting in substantial fluorescence enhancements^44^. Similarly, the results were substantiated by the activity and localization of the large molecular weight FITC dextran and provided an intrinsic means to examine microarchitecture integrity. Finally, the infusion of saline solution was used as a negative control during the imaging process.

IVM micrographs were collected using FV10-ASW Viewer 3.1 software (Olympus Corporation, Shinjuku, Tokyo, Japan), and the raw data exported as images in Tag Image File Format (TIFF) that were analyzed using ImageJ (Fiji- ImageJ 64, US National Institutes of Health, Bethesda, MD, USA) software packages. Upon curation of the data, all biochemical data was logged into an Excel file for analysis. All the logged data were reviewed to ensure no data entry errors occurred before analyses were conducted.

## Usage Notes

Data processing and analysis procedures, and the results for biochemical assays (UV-visible spectroscopy DNA and SDS concentration measurements), and IVM assays (DNA, cell, and tissue fluorescent intensity measurements, and microvascular leakage, and tubular and peritubular integrity) are presented in our repository in the file called IVM_usage_notes.csv. This file outlines specific details on the ways the data was handled. Our group and others in the field have previously used biochemical assays to analyze decellularization efficacy. Moreover, the IVM analyses performed have been adapted from related evaluations conducted by our group and several others. In both cases, the respective citations have been outlined. The repository is publicly available under a Creative Commons CCO license to support further analysis and publication under the requirement of citing this article and the dataset.

## Data Availability

No custom code was used to generate or process the data.
